# Experience of weekly cisplatin concurrent with intensity-modulated radiotherapy for locally advanced nasopharyngeal carcinoma patients with resistance to neoadjuvant chemotherapy

**DOI:** 10.1097/MD.0000000000008434

**Published:** 2017-11-03

**Authors:** Chuanben Chen, Taojun Chen, Chaoxiong Huang, Jing Wang, Zhaodong Fei

**Affiliations:** a Department of Radiation Oncology, Fujian Provincial Cancer Hospital, Teaching Hospital of Fujian Medical University; b Provincial Clinical College, Fujian Medical University; c Department of Radiation Oncology, Cancer Hospital of Fujian Medical University, Fuzhou, Fujian, People's Republic of China.

**Keywords:** concurrent chemoradiotherapy, intensity-modulated radiotherapy, nasopharyngeal carcinoma, neoadjuvant chemotherapy, therapeutic resistance, weekly cisplatin

## Abstract

Nasopharyngeal carcinoma (NPC) is highly sensitive to radiotherapy. Locally advanced NPC has a relatively poor prognosis if treated with radiotherapy alone. Several studies have demonstrated that chemoradiotherapy confers survival benefit in locally advanced NPC. However, a small proportion of patients are resistant to chemotherapy based on cisplatin. So, it is important to make a valuable and inexpensive schedule for these patients. After 2 cycles of neoadjuvant chemotherapy that consisted of gemcitabine and cisplatin (80 mg/m^2^, every 3 weeks) or paclitaxel and cisplatin (80 mg/m^2^, every 3 weeks), magnetic resonance imaging (MRI) was used to evaluate efficacy. A total of 13 patients with extensive nodal disease or/and bulky tumors volume were determined with a stable disease (SD) and enrolled in this study. Cisplatin at a dose of 30 mg/m^2^ administered weekly concurrent with intensity-modulated radiotherapy (IMRT) was used to treat these patients resistant to neoadjuvant chemotherapy. The efficacy was evaluated by tumor response and the change of tumor volume. After the completion of concurrent chemoradiotherapy (CCRT), the overall tumor response was a complete response (CR) for 4 of 13 (30.8%) patients and partial response (PR) for 9 of 13 (69.2%) patients. The mean primary tumor volume was reduced by 59.7% and 89.8% at the 24th fraction of IMRT and after the completion of IMRT, respectively. The mean nodal volume was reduced by 63.8% and 93.5% at the 24th fraction of IMRT and after completion of IMRT, respectively. The study showed that weekly cisplatin concurrent with IMRT improved the treatment parameters for locally advanced NPC with resistance to neoadjuvant chemotherapy based on cisplatin. It was a valuable and relatively inexpensive schedule to improve the prognosis for these patients.

## 1 Introduction

Nasopharyngeal carcinoma (NPC) is most prevalent in Southern China.^[1]^ Due to high radiation sensitivity, radiation therapy (RT) is the mainstay of treatment for early-stage NPC. However, according to the 7th Edition of the American Joint Commission on Cancer (AJCC) staging system,^[2]^ 60% to 70% of patients present with stage III–IV disease at initial diagnosis.^[3]^ Locally advanced NPC has a relatively poor prognosis if treated with RT alone. Due to the apparent chemosensitivity of the disease, chemotherapy (neoadjuvant, adjuvant, or concurrent) has been added to the treatment regimen for NPC in numerous randomized studies in an attempt to improve the outcome of locally advanced NPC patients.

Several studies have consistently demonstrated that concurrent chemoradiotherapy (CCRT) confers survival benefit in locally advanced NPC.^[4–12]^ Cisplatin is considered as a standard chemotherapy regimen of CCRT. Nevertheless, CCRT may not be adequate for certain high-risk patient groups, especially patients with extensive nodal disease or bulky tumors volume. Adding neoadjuvant chemotherapy might be a reasonable approach in such patients. Neoadjuvant chemotherapy may offer the benefits of reduction of tumor burden, which result in tumor hypoxia and resistance of radiation.

At our center, neoadjuvant chemotherapy plus CCRT is the standard treatment for locally advanced NPC patients with extensive nodal disease and/or bulky tumors volume. Neoadjuvant chemotherapy before RT may reduce the tumor burden, decrease planned target volume, and improve the complete remission rate. The reported rates of overall CR, PR, and SD after neoadjuvant chemotherapy range from 8% to 27%, 55% to 64%, and 11% to 17.6%, respectively.^[13–16]^ A small proportion of patients are resistant to neoadjuvant chemotherapy based on cisplatin. The change on the tumor burden did not differ significantly after neoadjuvant chemotherapy. These patients often have residual tumor after CCRT and higher potential for metastasis. Previous studies reported that the unsatisfactory tumor response after neoadjuvant chemotherapy could predict poor prognosis for patients with advanced NPC.^[17]^ Choosing a suitable treatment is necessary for these patients. We performed this study to evaluate the significance of weekly cisplatin and IMRT for locally advanced NPC patients with resistance to neoadjuvant chemotherapy based on cisplatin. To our knowledge, the subject has not been previously explored.

## 2 Patients and methods

### 2.1 Patients selection

Before treatment, all patients underwent a complete physical examination and medical history review, including nasopharyngoscopy and biopsy, full blood count, comprehensive serum chemistry profile, computed tomography (CT), electrocardiogram, ultrasonography of the abdomen, bone scan, and a magnetic resonance imaging (MRI) scan of the nasopharynx and neck. After 2 cycles of neoadjuvant chemotherapy, all patients underwent MRI scans of the nasopharynx and neck. On the basis of the Response Evaluation Criteria in Solid Tumors (RECIST),^[18]^ patients determined SD would be enrolled in the study. To ensure safety and smooth of the process, all patients included the study were in good general health and there were no severe complications during the course of neoadjuvant chemotherapy. A total of 13 NPC patients with extensive nodal disease or/and bulky tumors volume were determined SD and enrolled in this study. The retrospective analysis of the patient data got approval from the ethics committee of Fujian Provincial Cancer Hospital. All patients provided written informed consent.

### 2.2 Chemotherapy

The neoadjuvant chemotherapy regimen comprised gemcitabine (1000 mg/m^2^, on day (d)1 and d8, every 3 weeks) and cisplatin (80 mg/m^2^, on d2, every 3 weeks) or Paclitaxel (135 mg/m^2^, on d1, every 3 weeks) and cisplatin (80 mg/m^2^, on d2, every 3 weeks) before the administration of radiotherapy. All patients completed 2 cycles of neoadjuvant chemotherapy. If necessary, the dose was modified according to interim toxicity effects and the nadir blood counts during the preceding cycle. If the platelet count decreased to ≤25,000/mL or the leukocyte count decreased to ≤1000/mL, the doses of drugs were reduced by 25% in the subsequent cycle.

For patients enrolled in the study, cisplatin at a dose of 30 mg/m^2^ was administered weekly starting from Week 1 to 7 consecutive weeks during the course of RT. Patients were hydrated with more than 1500 mL of normal saline per session. Administration of cisplatin with RT was interrupted when patients developed a white blood cell (WBC) count < 3000/mL, or Grade ≥3 nonhematological toxicities (e.g., emesis, mucositis, fatigue), or serum creatinine >1.5 mg/dL. For prevention of emetics, 5-HT3 receptor antagonists and dexamethasone were given with the chemotherapy.

### 2.3 Radiotherapy

All patients in this study were treated with IMRT. Philips Pinnacle9.2 (Pinnacle, version 9.2, Philips Radiation Oncology System, Wisconsin) was used to design the IMRT plans. Treatment planning was performed with Elekta Synergy VMAT (Elekta Oncology System, Crawly, UK) linear accelerators. The target volumes were delineated using an institutional treatment protocol defined as follows. The primary gross tumor volume (GTV-P) and the involved lymph nodes (GTV-N) included all gross disease as determined by imaging, clinical, and endoscopic findings. The clinical target volumes (CTV-1, CTV-2) represented tissues felt to harbor the risk of microscopic disease. The CTV-1 was defined as the high-risk region that included GTV and 5 to 10 mm margin, including the nasopharyngeal mucosa (5 mm submucosal volume). The CTV-2 was designed for potentially involved regions, including the nasopharyngeal cavity, maxillary sinus, pterygopalatine fossa, posterior ethmoid sinus, parapharyngeal space, skull base, anterior third of clivus and cervical vertebra, inferior spheniod sinus, and cavernous sinus. Levels II–V can be incorporated into clinical target volume of the neck nodal regions (CTV-N), as recommended by the Radiation Therapy Oncology Group (RTOG) delineation consensus for head and neck malignancies. The planning target volume was created on the basis of each volume with an additional 3-mm margin, allowing for setup variability. OAR include the brain stem, spinal cord, optic nerve, optic chiasm, temporal lobe, crystal, and parotid, pituitary and mandibular glands, and so on. A total dose of 69.7 Gy in 34 fractions at 2.05 Gy/fraction to the planning target volume of GTV-P and GTV-N, 61.2 Gy at 1.8 Gy/fraction to the planning target volume of CTV-1, 54.4 Gy at 1.6 Gy/fraction to the planning target volume of CTV-2, and CTV-N were prescribed.

### 2.4 Evaluating indicator

Tumor response was acquired at the beginning and the end of neoadjuvant chemotherapy, the 24th fraction of subsequent IMRT, and the completion of IMRT by using MRI. Evaluation Criteria in Solid Tumors (RECIST).^[18]^ Complete response (CR) was defined as disappearance of target lesion (short radius of target neck pathological lymph nodes < 10 mm). Partial response (PR) was defined as a reduction of at least 30% in the sum of longest diameter of target lesions (using baseline sum longest diameter as reference). Progressive disease (PD) was defined as an increase of at least 20% in the sum of longest diameter of target lesions (using smallest sum longest diameter since treatment started or appearance of one or more new lesions as reference). Stable disease (SD) was defined as neither sufficient shrinkage to qualify for PR nor sufficient increase to qualify for PD (using smallest sum longest diameter since treatment started as reference). Overall tumor response, local tumor response, and regional lymph node response are defined in Table 1.

**Table 1:**
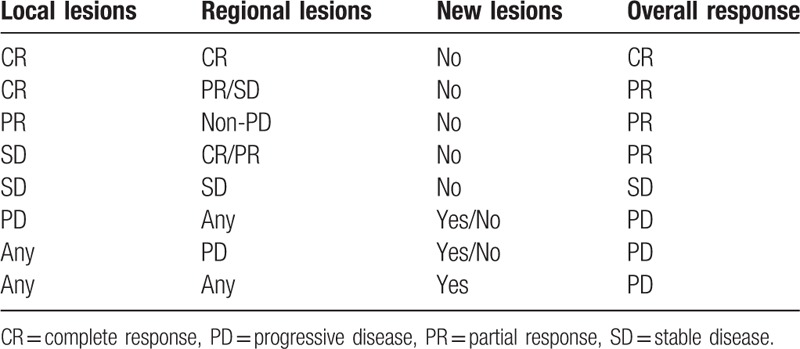
Tumor response for treatment.

MRI data were imported in a 3D treatment-planning system and a radiation oncologist manually outlined the primary lesion of each image. The tumor volume can be automatically calculated by reconstructing a 3D image. The primary tumor and lymph node volume were recorded in turn. If 1 patient had bilateral nodes metastases, the volume would be independently assessed. Results were analyzed and quantified with SPSS software (SPSS Inc, Chicago, IL).

### 2.5 Statistical analysis

All data were analyzed using SPSS 17.0 statistical software (SPSS Inc, Chicago, IL). A repeated-measures analysis of variance (ANOVA) was used to calculate the reduction of tumor volume at different time points (the initiation and end of 2 cycles of neoadjuvant chemotherapy, the 24th fraction of subsequent IMRT, and the completion of IMRT). Statistical significance was accepted as a *P* value < .05.

## 3 Results

### 3.1 Patient characteristics

After 2 cycles of neoadjuvant chemotherapy, all patients underwent MRI scans of the nasopharynx and neck. A total of 13 NPC patients with extensive nodal disease or/and bulky tumors volume determined SD were enrolled in the study. Patients’ characteristics are displayed in Table 2.

**Table 2:**
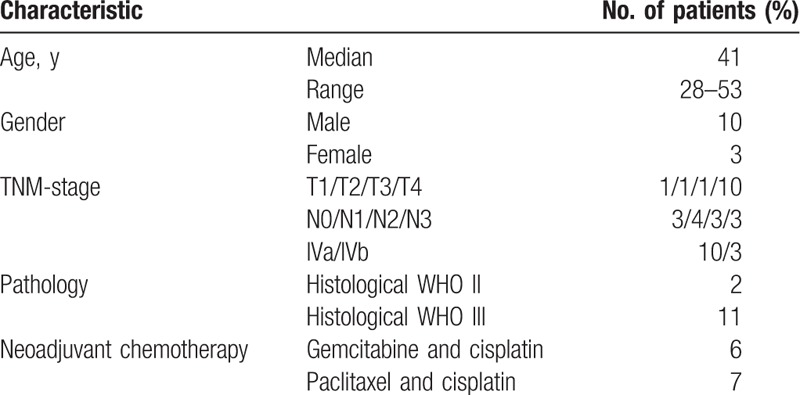
Patients’ characteristics.

### 3.2 Assessment of tumor response

For overall tumor response after completion of IMRT, 4 patients (30.8%) achieved CR, while the rest 9 patients (69.2%) showed PR. No patients had SD or PD. One of 4 patients already showed CR by MRI at the 24th fraction of IMRT. Tumor response is listed in Table 3.

**Table 3:**
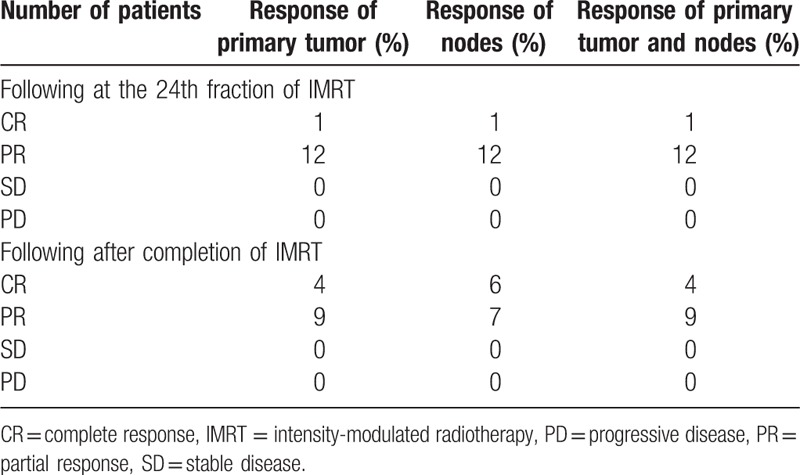
Assessment of tumor response.

### 3.3 Assessment of tumor volume

**Figure 1:**
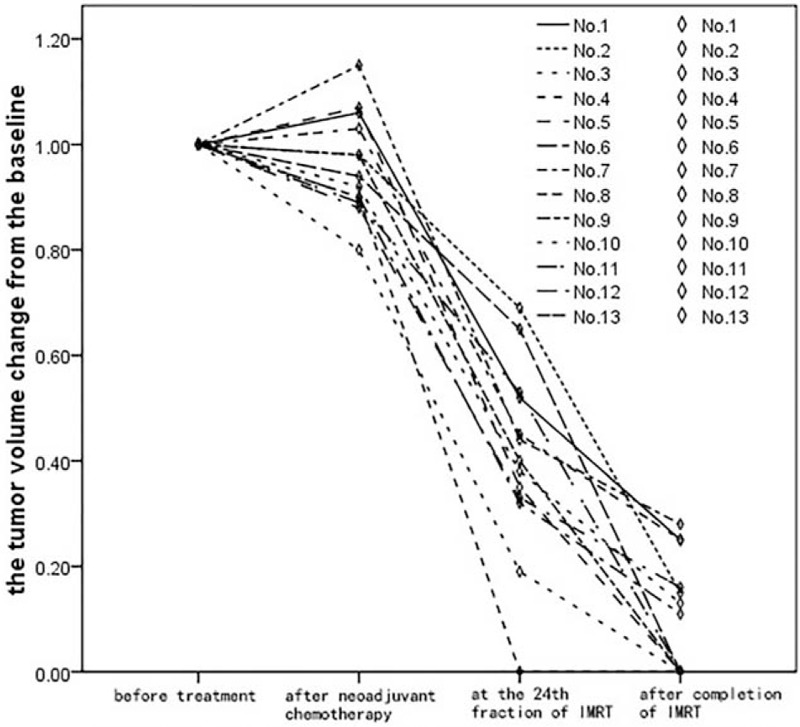
The reduction of tumor volume during treatment.

**Figure 2:**
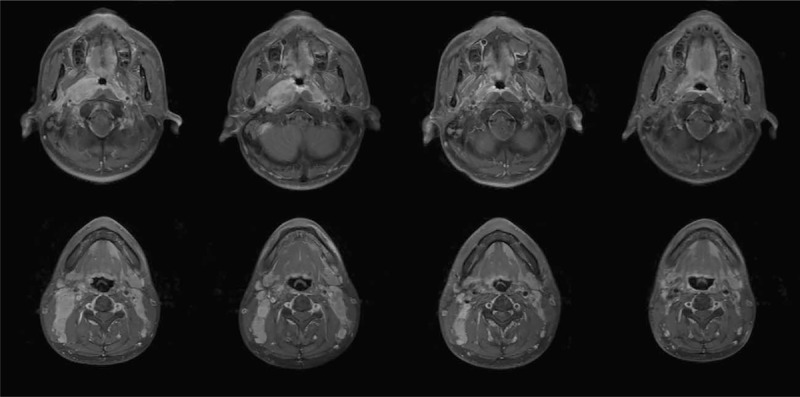
The same MR cross-sectional imaging at different time points of the same patient (before treatment, after 2 cycles of neoadjuvant chemotherapy, at the 24th fraction of IMRT, and after completion of IMRT).

For baseline comparison, the tumor volume before treatment were defined as 100%. The reduction of tumor volume was calculated relative to the volume before treatment. The mean of primary tumor volume for all NPC patients was 39.8 ± 18.1(SD) mL with the range of 6.1 to 66.4 mL. And the mean of the nodal volume was 22.9 ± 17.8 (SD) mL with the range of 5.1 to 77.3 mL. It should be noted that the data were calculated separately if 1 patient has bilateral lymph node metastases. The mean primary tumor volume was reduced by 59.7% and 89.8% at the 24th fraction of IMRT and after completion of IMRT, respectively (*P* < .001). The mean nodal volume was reduced by 63.8% and 93.5% at the 24th fraction of IMRT and after completion of IMRT, respectively (*P* < .001). Figure 1 shows the volumetric reduction of primary tumor. Figure 2 shows the same MR cross-sectional imaging at different time points of the same patient.

### 3.4 Toxicity

The acute toxicities experienced during CCRT can be tolerated. There was no incidence of treatment-related mortality, and the most frequent toxicities were neutropenia, oral mucositis, and xerostomia. Toxicities are listed in Table 4 during CCRT.

**Table 4:**
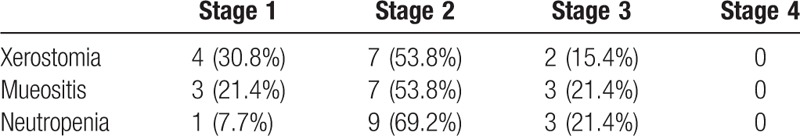
Acute toxicity of concurrent chemoradiotherapy.

## 4 Discussion

The prognosis of patients treated with radiotherapy alone for stage III-IVb NPC is poor. Radiotherapy combined with chemotherapy have shown the potential to improve the prognosis. Neoadjuvant chemotherapy and CCRT is the standard treatment for locally advanced NPC patients with extensive nodal disease and/or bulky tumors volume at our center. Neoadjuvant chemotherapy can shrink the primary tumor or even significantly down stage, resulting in a wider distance from irradiation field margin and better protection of important normal tissues, such as critical neurological structures. A small proportion of patients are resistant to neoadjuvant chemotherapy based on cisplatin. So, neoadjuvant chemotherapy followed by triweekly delivery of cisplatin concurrent with a course of RT would not be appropriate for these patients. Previous studies reported that unsatisfactory tumor response after neoadjuvant chemotherapy could predict poor prognosis for patients with advanced NPC.^[17]^ Recently, a retrospective analysis was reported on the survival of 412 consecutive patients with stage III-IVb NPC treated with neoadjuvant chemotherapy followed by triweekly delivery of cisplatin concurrent with RT. The study confirmed that patients whose tumor shrank insignificantly after neoadjuvant chemotherapy have a worse prognosis compared with patients whose tumor shrank significantly.^[19]^

Previous research had revealed that cells in large tumors are less sensitive both to radiation and to cytotoxic drugs than cells in small nodules. The tumor of more than a certain diameter will lack oxygen especially in the center of the mass.^[20]^ Radiosensitizers are intended to enhance the ability to kill tumor cell while having less effect on normal tissue. Some drugs target different physiological characteristics of the tumor, particularly hypoxia associated with radioresistance. Cetuximab are proven to enhance radiosensitivity in NPC.^[21,22]^ Unfortunately, Cetuximab are not only unaffordable for many patients in developing countries but also have limited value in clinical practice.

Our study showed that weekly cisplatin concurrent with IMRT improves the treatment parameters for locally advanced NPC with resistance to neoadjuvant chemotherapy, which is based on cisplatin. For overall tumor response after completion of IMRT, 4 patients (30.8%) achieved CR, while the rest 9 patients (69.2%) showed PR. The mean primary tumor volume was reduced by 59.7% and 89.8% at the 24th fraction of IMRT and after completion of IMRT, respectively. The mean nodal volume was reduced by 63.8% and 93.5% at the 24th fraction of IMRT and after completion of IMRT, respectively. The short-term efficacy after completion of CCRT is consistent with previous reports that aimed to evaluate the efficacy of neoadjuvant chemotherapy followed by chemoradiotherapy in locally advanced NPC.^[23]^ Weekly cisplatin concurrent with IMRT improves the treatment parameters for locally advanced NPC with resistance to neoadjuvant chemotherapy, which is based on cisplatin.

It is important to find a suitable schedule to improve the prognosis of patients who are resistant to neoadjuvant chemotherapy based on cisplatin. Previous studies have shown that low to moderate dose of external irradiation can reduce the tumor cells resistance to chemotherapy.^[24,25]^ Cisplatin has a radiosensitizing effect on radiation-resistant cells, including glioma cells known to have proficient repair, and human ovarian carcinoma cells, which are cisplatin resistant and cross-resistant to radiation.^[26,27]^ Weekly administration of cisplatin is based on the hypothesis that cisplatin can act as a radiosensitizor when it is used in a smaller dose and administered more frequently. Cisplatin as a chemotherapeutic agent induces DNA changes in malignant cells that may be mutagenic or lethal. When used concurrently with RT, cisplatin acts as a radiosensitizer, increasing damage to nuclear DNA from malignant cells to enhance the anti-neoplastic capability of radiotherapy. The mechanism of this radiosensitization may involve cisplatin's ability to inhibit sublethal damage repair in radiated tumor cells. Weekly administration of cisplatin maintains a constant stimulation and enhances the tumor cells sensitivity to radiotherapy.

There are limitations in the present study. As an exploratory study, the total amount of patients enrolled in was relatively small. Patients’ baseline health characteristics were restricted in the study. All patients enrolled in the study were in good general health and ages ranged from 28 to 53 years. There were no severe complications during the course of neoadjuvant chemotherapy. The general health assessments indicate that CCRT can be tolerated and the program was expected to be completed. Some studies showed that once-weekly cisplatin combined with radiation for the treatment of locally advanced NPC has parallel efficacy and safety compared with a triweekly regimen.^[28]^

Poor reduction of tumor volume after neoadjuvant chemotherapy was a predictor of adverse outcomes. Our study suggested that weekly cisplatin concurrent with IMRT improves the treatment parameters for locally advanced NPC with resistance to neoadjuvant chemotherapy based on cisplatin. It was a suitable schedule to improve the prognosis for these patients. Weekly administration of cisplatin maintains a constant stimulation and enhances the tumor cells sensitivity to radiotherapy likely serves as radiosensitizer. Additional work is required to elucidate the detailed mechanism.
